# Mapping intrinsic electromechanical responses at the nanoscale via sequential excitation scanning probe microscopy empowered by deep data

**DOI:** 10.1093/nsr/nwy096

**Published:** 2018-09-08

**Authors:** Boyuan Huang, Ehsan Nasr Esfahani, Jiangyu Li

**Affiliations:** 1 Department of Mechanical Engineering, University of Washington, Seattle, WA 98195-2600, USA; 2 Shenzhen Key Laboratory of Nanobiomechanics, Shenzhen Institutes of Advanced Technology, Chinese Academy of Sciences, Shenzhen 518055, China

**Keywords:** sequential excitation, scanning probe microscopy, principal component analysis, simple harmonic oscillator model

## Abstract

Ever-increasing hardware capabilities and computation powers have enabled acquisition and analysis of big scientific data at the nanoscale routine, though much of the data acquired often turn out to be redundant, noisy and/or irrelevant to the problems of interest, and it remains nontrivial to draw clear mechanistic insights from pure data analytics. In this work, we use scanning probe microscopy (SPM) as an example to demonstrate deep data methodology for nanosciences, transitioning from brute-force analytics such as data mining, correlation analysis and unsupervised classification to informed and/or targeted causative data analytics built on sound physical understanding. Three key ingredients of such deep data analytics are presented. A sequential excitation scanning probe microscopy (SE-SPM) technique is first developed to acquire high-quality, efficient and physically relevant data, which can be easily implemented on any standard atomic force microscope (AFM). Brute-force physical analysis is then carried out using a simple harmonic oscillator (SHO) model, enabling us to derive intrinsic electromechanical coupling of interest. Finally, principal component analysis (PCA) is carried out, which not only speeds up the analysis by four orders of magnitude, but also allows a clear physical interpretation of its modes in combination with SHO analysis. A rough piezoelectric material has been probed using such a strategy, enabling us to map its intrinsic electromechanical properties at the nanoscale with high fidelity, where conventional methods fail. The SE in combination with deep data methodology can be easily adapted for other SPM techniques to probe a wide range of functional phenomena at the nanoscale.

## INTRODUCTION

The fusion of scientific research and big data has provided an unprecedented opportunity for accelerated discovery, understanding and innovation [1–6], yet it also imposes new challenges for scientists to adjust to, adapt to and thrive in the face of daunting data volume [7,8]. Ever-increasing hardware capabilities and computation powers have made acquisition and analysis of big scientific data routine, though much of the data acquired often turn out to be redundant, noisy and/or irrelevant to the problems of interest, and it remains nontrivial to draw clear mechanistic insights from brute-force data analytics [2,7,9]. As such, there is strong desire to push big data toward deep data, namely from data mining, correlation analysis and unsupervised classification to causative data analytics that fuse scientific knowledge of physics, chemistry and biology into big data [2,3,5,6,9,10] and thus from brute forces to informed and/or targeted strategies. We believe that three key questions need to be answered to enable such a vision: (i) how do we develop innovative experimental and/or computational methodologies to acquire high-quality (less noisy), efficient (less redundant) and physically relevant scientific data to enable deep analysis; (ii) how can we learn clear mechanistic insights from the data, guided by the underlying physical principles; and (iii) how can we accelerate and enhance physical understanding by informed and targeted big-data analytics? Scanning probe microscopy (SPM) is capable of acquiring multi-dimensional physical datasets in the form of high-resolution images and spectroscopy [11–14], providing us with an ideal playground for deep data methodology. In this work, we demonstrate the essence of such deep data analysis via a simple yet revealing case study of SPM that incorporates all the three key ingredients above, wherein an experimental methodology is designed to acquire high-quality, efficient and physically relevant SPM data amenable to both physical modeling and data analytics, making it possible to accelerate and enhance physical understanding by targeted big data. This also enables us to resolve a long-standing challenge in scanning probe microscopy—mapping weak intrinsic responses at the nanoscale quantitatively [15–17]. Of particular interest is a clear mechanistic understanding of the unsupervised principle component analysis (PCA) [18–20] acquired through sound physical principle, which speeds up physical analysis by at least four orders of magnitude.

## RESULTS AND DISCUSSION

### Sequential excitation

**Figure 1.**
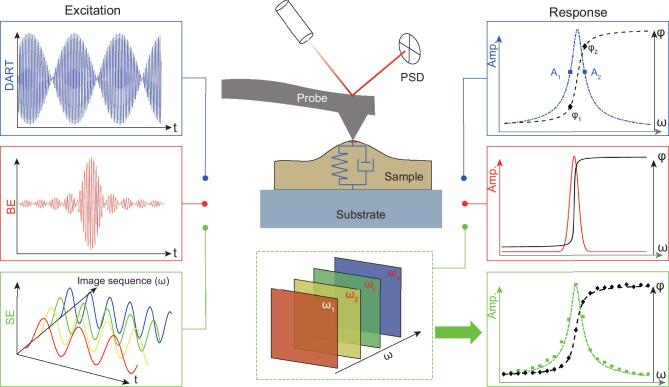
The schematics of dynamic SPM experiments based on DART, BE and SE techniques, wherein the AC waveforms combining two distinct or a band of frequencies are synthesized to excite the sample under DART or BE, respectively, while a sequence of AC waveforms with different frequencies are used to excite the sample under SE.

As the first step, we develop a sequential excitation scanning probe microscopy (SE-SPM) technique [21] to acquire high-quality, efficient and physically relevant data in the frequency domain, and the method can be easily implemented in any standard atomic force microscope (AFM) without the need for any additional hardware and instrumentation [22]. To this end, we note that the majority of SPM measurements deduce physical properties of samples from the interactions between a cantilever with a sharp tip and a sample surface, as schematically shown in Fig. 1, and the dynamics of the interaction can be described well by a simple harmonic oscillator (SHO) model [23,24]:

(1.1) \begin{equation*} A(\omega ) = \frac{{{A_0}\omega _0^2}}{{\sqrt {{{(\omega _0^2 - {\omega ^2})}^2} + {{\left( {\frac{{{\omega _0}\omega }}{Q}} \right)}^2}} }}{\rm{,}} \end{equation*}

(1.2) \begin{equation*} \phi (\omega ) = {\tan ^{ - 1}}\left[ {\frac{{{\omega _0}\omega }}{{Q(\omega _0^2 - {\omega ^2})}}} \right], \end{equation*}

where ${A_0}$, ${\omega _0}$, and $Q$ are intrinsic electromechanical response (piezoelectricity), resonant frequency (elasticity) and quality factor (energy dissipation) of the system that we are interested in determining quantitatively from the SPM experiment, not only at a single point, but over a spatial mapping. Note that (*ω*), is the excitation frequency, with $A(\omega )$ and $\phi (\omega )$ as the corresponding amplitude and phase that are directly measured in experiment. As such, it is desirable to acquire the amplitude $A(\omega ,x,y)$ and phase $\phi (\omega ,x,y)$ over a 2D space $(x,y)$ of the sample surface as well as a frequency spectrum of excitation (*ω*), from which ${A_0}$, ${\omega _0}$, and $Q$, the physical parameters of interest, can be solved by fitting Equation (1). Conventional techniques such as dual amplitude resonance tracking (DART) [24,25] and band excitation (BE) [26,27] synthesize the AC waveform, combining either two distinct or a band of frequencies to excite the sample (Fig. 1). The former yields only two sets of data, not amenable for reliable fitting of the highly non-linear Equation (1), and the resonance tracking is not always robust, especially for rough sample surfaces. The latter is capable of capturing the complete spectrum without resonance tracking, though it distributes excitation energy over a frequency band, reducing its signal strength substantially, and thus the data quality is not high. Newly developed general mode (G-mode) SPM records complete time- instead of frequency-domain data that are very powerful [7,28], although a substantial portion of the data could be redundant and thus the subsequent analysis is not very efficient, and it requires sophisticated instrumentation that is not easily accessible. Therefore, there is still a strong desire for an innovative yet simple and easily accessible approach to acquire high-quality (less noisy), efficient (less redundant) and physically relevant SPM data to enable deep analysis.

To this end, we developed sequential excitation (SE) that excites the sample using a sequence of AC waveforms with distinct frequencies ${\omega _j}$, as shown in Fig. 1, wherein the excitation energy is concentrated on only one frequency at a time instead of being distributed over a band of spectra, ensuring that the signal is strong and the response is not noisy. In such a setup, each excitation frequency captures cantilever-sample resonance at selected spatial points that are unique, ensuring that the data are relevant yet not redundant. Furthermore, no resonance tracking is needed as in DART, ensuring that the measurement is robust and reliable. In a sense, such a strategy of SE is analogous to super-resolution microscopy in biology that turns specific fluorescent molecules on and off in a sequential manner for imaging [29,30], wherein we excite specific resonances of different points sequentially using distinct frequencies. Our approach, however, requires no extra hardware and further instrumentation in a standard AFM, and it can be easily implemented, making it widely accessible.

**Figure 2.**
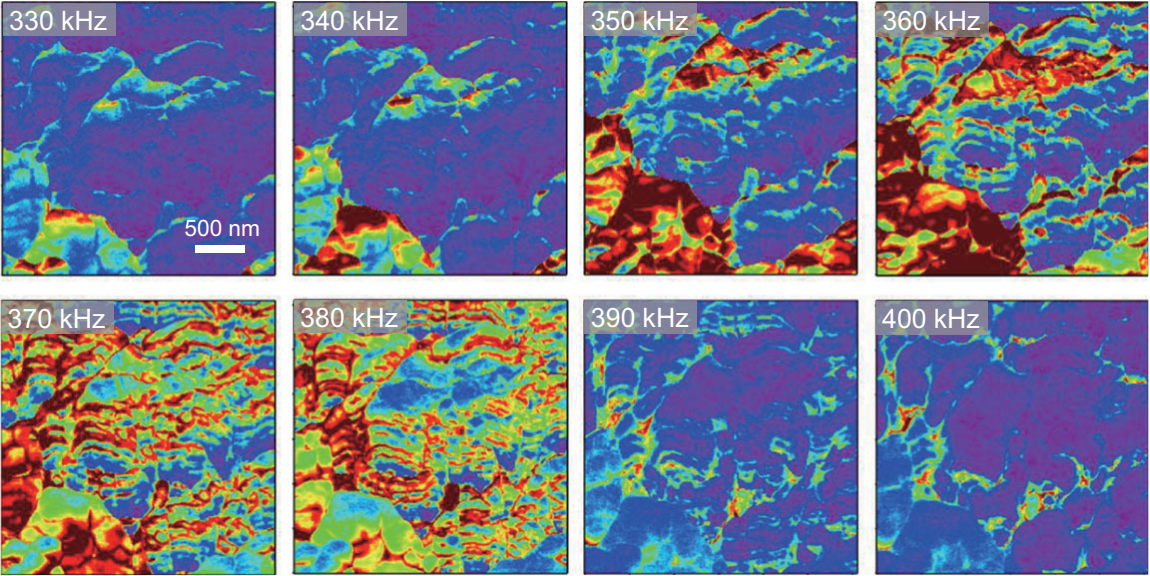
A sequence of SE-PFM amplitude mappings obtained at distinct frequen-cies in PZT ceramic.

As a demonstration, we studied the piezoresponse force microscopy (PFM) of a PZT ceramic under SE, wherein a sequence of its amplitude mappings $A({\omega _j},x,y)$ are shown in Fig. 2, obtained using AC excitation frequencies ranging from 320 to 400 kHz as determined from a preliminary DART scan. The drifting between different scans has been corrected as detailed in Supporting Information (SI), available as Supplementary Data at *NSR* online. It was observed that the PFM amplitude is very sensitive to the excitation frequency ${\omega _j}$, as expected, and there are substantial amplitude changes when the excitation frequency varies. In addition, substantial spatial heterogeneity is observed within each mapping, reflecting possible variations in intrinsic piezoelectricity, elasticity, energy dissipation or their combinations. Such crosstalk makes it difficult to determine the intrinsic SPM response quantitatively, and it is necessary to deconvolute these different effects. In fact, our work was originally motivated by this very issue, which has important implications in the nanoscale probing of electromechanical coupling, ubiquitous in nature, that underpins the functionalities of both synthetic materials and the biology for information processing as well as energy conversion and storage [11,31–37]. While dynamic strain-based SPM techniques have emerged as a powerful tool to investigate electromechanical coupling at the nanoscale in the last decade [11], which are known as PFM for piezoelectrics and ferroelectrics [36,38–44] and as electrochemical strain microscopy (ESM) for electrochemical systems [45–50], determining intrinsic electromechanical response remains challenging due to its crosstalk with topography, elasticity and energy dissipation. SE-PFM makes it possible to overcome such difficulties, though we must reconstruct data to determine the intrinsic response, analogous to super-resolution microscopy in biology. In this regard, the 3D data sets of $A(\omega ,x,y)$ and phase $\phi (\omega ,x,y)$ obtained from SE are amenable to both physics-based SHO analysis and statistics-based PCA, making deep data analysis possible.

### Physical analysis by a simple harmonic oscillator model

**Figure 3.**
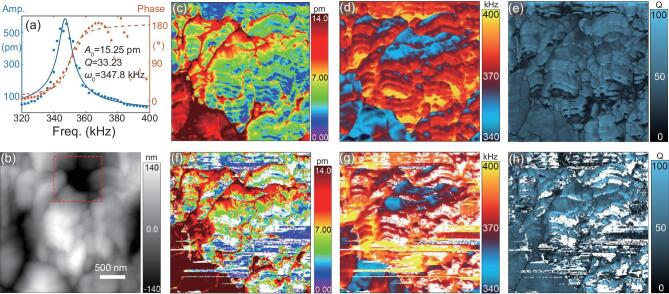
Comparison of PZT mappings acquired by SE-PFM and DART-PFM processed via SHO; (a) SHO fitting of SE-PFM spectrum data for one representative pixel; (b) rough topography mapping; and (c)–(e) reconstructed SE-PFM mappings of (c) intrinsic amplitude ${A_0}$, (d) resonance frequency ${\omega _0}$ and (e) quality factor $Q$; (f)–(h) reconstructed DART-PFM mappings of (f) the intrinsic amplitude, (g) resonance frequency and (h) quality factor obtained, wherein white areas show points where SHO analysis fails.

We start with brute-force physical analysis accomplished by fitting 3D datasets of amplitude $A(\omega ,x,y)$ at each pixel $(x,y)$ using the SHO model of Equation (1.1). This is demonstrated by one representative pixel in Fig. 3a, yielding a resonant frequency of 347.8 kHz, quality factor of 33.23 and intrinsic electromechanical response of 15.25 pm at that particular point. Note that the sample surface is rather rough, as revealed by its topography (Fig. 3b), which imposes substantial difficulty for DART-PFM, yet SHO analysis can be easily applied to each pixel of the SE-PFM to reconstruct the mappings of intrinsic amplitude, resonant frequency and quality factor, as shown in Fig. 3c–e. Indeed, there is strong spatial variation in the intrinsic amplitude mapping, though little correlation is seen between topography (Fig. 3b) and amplitude (Fig. 3c), even in regions with substantial roughness, such as in the valley on the top part of the mapping marked by the dotted red square. The mapping of *R*^2^—a statistical measure known as the fitting coefficient of determination assessing how close the data are to the fitted regression line—is presented in Supplementary Fig.  1 in SI, available as Supplementary Data at *NSR* online, revealing values ranging from 0.85 to 0.99 and thus a high fidelity of SHO analysis. This demonstrates the capability of SE-PFM even for highly inhomogeneous and rough samples. Such a capability, however, is beyond the conventional DART-PFM, as exhibited in Fig. 3f–h, where it is observed that a significant percentage of points (27%) fail to yield a valid solution in SHO analysis, as highlighted by the white pixels in the mappings. Such a problem also casts doubts on the points wherein SHO analysis works and, indeed, mappings of intrinsic amplitude, resonant frequency and quality factors all show not so subtle difference between SE- and DART-PFM, especially in rough regions. This highlights the advantage of SE over DART, which measures responses at only two excitation frequencies across resonance, as schematically shown in Fig. 1, and uses the difference between these two responses as feedback for resonance tracking. Such a strategy often runs into difficulties: if the separation between two excitation frequencies is too small, they will easily fall out of resonance range during scanning and thus fail to track resonance shift; and, if the separation is too large, then the responses are weak and the signal-to-noise ratios are low. For materials exhibiting substantial heterogeneity at the nanoscale, for instance near the grain boundaries [16,51,52] wherein the contact resonance frequency can shift significantly over a relatively short distance, resonance tracking and thus SHO analysis often fail [17]. This is clearly demonstrated in PZT (Fig. 3f–h), which has strong piezoelectric response yet a rough topography (Fig. 3b) that is not uncommon in practice, and the excitation frequency must shift substantially during scanning (Fig. 3g and Supplementary Fig. 2, available as Supplementary Data at *NSR* online). An excellent ferroelectric material such as PZT still suffers from such a difficulty, and the issue is all the more serious for other materials with weaker electromechanical coupling. Mappings acquired from SE-PFM, on the other hand, are free of such problems.

### PCA and its physical interpretation

While SHO fitting works well under SE-PFM, it is a computationally expensive process, taking an Intel Xeon E5–2695 CPU approximately 0.06 s for one pixel and thus 1.09 hr for a 256 × 256 mapping (or 5.8 min for a parallel pool with 28 CPU workers). We thus resort to multivariate statistical tools such as PCA to speed up the analysis [18], as a sequence of images has been obtained under different excitation frequencies, ideal for PCA. Through orthogonal transformation, PCA converts a set of possibly correlated variables, in this case SE-PFM mappings under different excitation frequencies, to a set of linearly uncorrelated variables known as principal components. As a powerful unsupervised data-analytic tool, PCA is widely used to compress and visualize multi-dimensional datasets, though its physical meaning is often unclear.

Here, we demonstrate that PCA not only speeds up our computation by four orders of magnitude, but also allows a clear physical interpretation of its modes in combination with SHO analysis. To this end, we recast a 3D dataset of $A(\omega ,x,y)$ into 2D dataset of $A(\omega ,x)$, where a 2D spatial grid is collapsed into 1D. This reshaped dataset can be viewed as a 2D matrix **A**, such that each row of **A** represents a spatial mapping at a particular excitation frequency, while each column represents a spectrum of data spanning all excitation frequencies for a particular grid point. The details of the following derivation are presented in the SI, available as Supplementary Data at *NSR* online wherein principal components of **A**, namely the spatial eigenvectors ${{{\bf w}}_i}$ of the covariance matrix **A**^T^**A**, are evaluated by a singular value decomposition (SVD) [53]. Therefore, the *j*^th^ row of **A**, **A***_j-row_*, can be regarded as a linear combination of ${{{\bf w}}_i}$ with coefficients ${\xi _i}$.

(2) \begin{equation*} {{{\bf A}}_{j\hbox{-} row}} = \sum\limits_i {{\xi _i}({\omega _j}\!{\rm{)}}} \cdot {{{\bf w}}_i}\!. \end{equation*}

On the other hand, **A***_j-row_* can also be reformulated from Equation (1) of SHO as detailed in SI, available as Supplementary Data at *NSR* online:

(3) \begin{equation*} {{{\bf A}}_{j\hbox{-} row}} = \sum\limits_i {{\lambda _i}({\omega _j}\!{\rm{)}}} \cdot {{{\bf \alpha }}_{{\bf i}}} = \sum\limits_i {{\zeta _i}({\omega _j}\!)} \cdot {{{\bf \beta }}_{{\bf i}}}, \end{equation*}

where components $\{ {{{\bf \alpha }}_{{\bf i}}}\} = {{{\bf A}}_{{\bf 0}}}{{\bf Q}}{{{\bf \omega }}_{{\bf 0}}} \circ {\rm{[}}{{\bf 1}}{\rm{, }}{{\bf Q}} - {{\bf \bar{Q}}}{\rm{, }} {{{\bf \omega }}_{{\bf 0}}} - {{{\bf \bar{\omega }}}_{{\bf 0}}}{\rm{, (}}{{{\bf \omega }}_{{\bf 0}}} - {{{\bf \bar{\omega }}}_{{\bf 0}}}{{\rm{)}}^2}{\rm{, }}...]$ inherit all spatial variance of vectors ${{{\bf A}}_{{\bf 0}}}$, ${{\bf Q}}$, and ${{{\bf \omega }}_{{\bf 0}}}$ that are reshaped from intrinsic parameter mappings ${A_0}(x,y)$, $Q(x,y)$, and ${\omega _0}(x,y)$, ${{\bf 1}}$ is a 1D vector with components to be 1, and ${{\bf \bar{Q}}} = {{\bf 1}} \cdot \bar{Q}$, ${{{\bf \bar{\omega }}}_{{\bf 0}}} = {{\bf 1}} \cdot {\bar{\omega }_0}$. Here, operator $\circ $ denotes the Hadamard product of two vectors, ${{{\bf A}}_{{\bf 0}}}{{\bf Q}}{{{\bf \omega }}_{{\bf 0}}} = {{{\bf A}}_{{\bf 0}}} \circ {{\bf Q}} \circ {{{\bf \omega }}_{{\bf 0}}}$, and the overhead bar is used to denote spatial averaging. Note that ${{{\bf \alpha }}_1}$ corresponds to ${{{\bf A}}_{{\bf 0}}}{{\bf Q}}{{{\bf \omega }}_{{\bf 0}}}$ because it is always the leading term in the 2D Taylor series, while the sequence of following ${{{\bf \alpha }}_i}$ depends on the relative variation of ${{\bf Q}}$ and ${{{\bf \omega }}_{{\bf 0}}}$, and the order could be different for different physical systems. Since ${{{\bf w}}_i}$ is orthogonal while ${{{\bf \alpha }}_{{\bf i}}}$ is not, to ensure the comparison with Equation (2), we transform $\{ {{{\bf \alpha }}_{{\bf i}}}\} $ into a set of orthonormal basis $\{ {{{\bf \beta }}_{{\bf i}}}\} $ via Gram–Schmidt process [54], with ${{{\bf \beta }}_1} = {{{\bf \alpha }}_1} = {{{\bf A}}_{{\bf 0}}}{{\bf Q}}{{{\bf \omega }}_{{\bf 0}}}$, ${{{\bf \beta }}_2} = {{{\bf \alpha }}_2} - \frac{{{{{\bf \alpha }}_2} \cdot {{{\bf \beta }}_1}}}{{{{\| {{{{\bf \beta }}_1}} \|}^2}}}{{{\bf \beta }}_1}$, and ${{{\bf \beta }}_3} = {{{\bf \alpha }}_3} - \frac{{{{{\bf \alpha }}_3} \cdot {{{\bf \beta }}_1}}}{{{{\| {{{{\bf \beta }}_1}} \|}^2}}}{{{\bf \beta }}_1} - \frac{{{{{\bf \alpha }}_3} \cdot {{{\bf \beta }}_2}}}{{{{\| {{{{\bf \beta }}_2}} \|}^2}}}{{{\bf \beta }}_2}$ after which $\{ {{{\bf \beta }}_{{\bf i}}}\} $ is normalized. The analogy between Equation (3) and Equation (2) is evident, suggesting that PCA components $\{ {{{\bf w}}_i}\} $ correspond to an orthonormal basis $\{ {{{\bf \beta }}_{{\bf i}}}\} $ derived from SHO, which has a clear physical interpretation related to intrinsic electromechanical response (piezoelectricity)${A_0}$, resonant frequency (elasticity) ${\omega _0}$, and quality factor (energy dissipation) $Q$ of the system. In a completely parallel manner, the correspondence between PCA spectral eigenvectors and SHO expansion can be established by switching the row and column of ${{\bf A}}$, namely between PCA spectral eigenvectors ${{\bf A}}{{{\bf w}}_i}$ and SHO spectral basis ${{\bf A}}{{{\bf \beta }}_i}$, as detailed in the SI, available as Supplementary Data at *NSR* online. In particular, elements of ${{\bf A}}{{{\bf w}}_i}$ represent the weight ${{{\bf \xi }}_i}$ that ${{{\bf w}}_i}$ takes up in each scan according to Equation (2).

**Figure 4.**
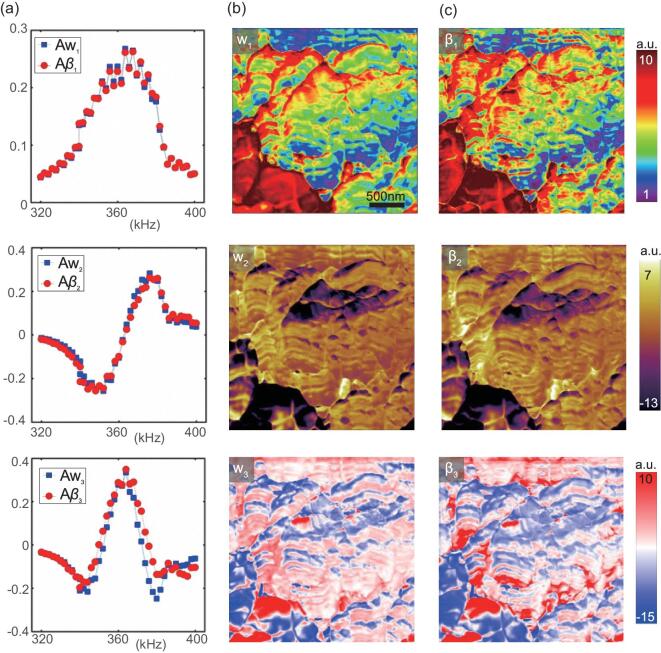
Comparison of PCA and SHO expansion for SE-PFM data of PZT; (a) first three PCA spectral eigenvectors in comparison with corresponding SHO spectral basis; (b) first three PCA spatial eigenvectors; (c) corresponding SHO spatial basis.

To demonstrate this analysis, we compare the first three spectral eigenvectors of PCA versus the SHO expansion in Fig. 4a, derived from SE-PFM data presented in Fig. 2. Good agreement between PCA spectral eigenvectors ${{\bf A}}{{{\bf w}}_i}$ and SHO spectral basis ${{\bf A}}{{{\bf \beta }}_i}$ is observed. The first three spatial eigenvectors of PCA are shown in Fig. 4b, in comparison with the first three SHO spatial basis in Fig. 4c, wherein good agreement is again observed, with ${{{\bf \beta }}_1} = {{{\bf A}}_{{\bf 0}}}{{\bf Q}}{{{\bf \omega }}_{{\bf 0}}}$, and ${{{\bf \beta }}_2}$ and ${{{\bf \beta }}_3}$ derived from ${{{\bf \alpha }}_2} = {{{\bf A}}_{{\bf 0}}}{{\bf Q}}{{{\bf \omega }}_{{\bf 0}}} \circ ({{{\bf \omega }}_{{\bf 0}}} - {{{\bf \bar{\omega }}}_{{\bf 0}}}{\rm{)}}$ and ${{{\bf \alpha }}_3} = {{{\bf A}}_{{\bf 0}}}{{\bf Q}}{{{\bf \omega }}_{{\bf 0}}} \circ {({{{\bf \omega }}_{{\bf 0}}} - {{{\bf \bar{\omega }}}_{{\bf 0}}}{\rm{)}}^2}$ via the Gram–Schmidt process. Note that, for the PZT sample probed, the second-order variation of ${{{\bf \omega }}_{{\bf 0}}}$ dominates the first-order variation of ${{\bf Q}}$, so that ${{{\bf \alpha }}_3} = {{{\bf A}}_{{\bf 0}}}{{\bf Q}}{{{\bf \omega }}_{{\bf 0}}} \circ {({{{\bf \omega }}_{{\bf 0}}} - {{{\bf \bar{\omega }}}_{{\bf 0}}}{\rm{)}}^2}$. The structural similarities (SSIMs) [55] for the first three pairs are evaluated to be 99.7, 98.5 and 95.5%, while the Pearson correlation coefficients (PCC) [56] are 90.06, 91.15 and 72.97%, as detailed in SI, available as Supplementary Data at *NSR* online, confirming our analysis numerically.

Intuitively, the set of SE-PFM mappings under different frequencies contains two important pieces of information: the variations of the amplitude with respect to the spatial locations and with respect to excitation frequencies, which are interconnected in the original mappings of Fig. 2. Under PCA, the data are transformed, such that the spatial variation is best represented by the spatial eigenvectors, and the frequency variation is reflected in the spectral eigenvectors for each PCA mode. Note that the principal components are sorted by their eigenvalues in a descending manner, with the first principal component accounting for the maximum possible variability in the data, as shown by the scree plot of variance in Supplementary Fig. 3, available as Supplementary Data at *NSR* online. The physical interpretations of PCA eigenvectors, however, are often unclear, which we have resolved in this work with the assistance of SHO analysis. Note that PCA takes only 0.24 s for an Intel Xeon E5–2695 CPU to complete, which is four orders of magnitude faster than brute-force fitting.

**Figure 5.**
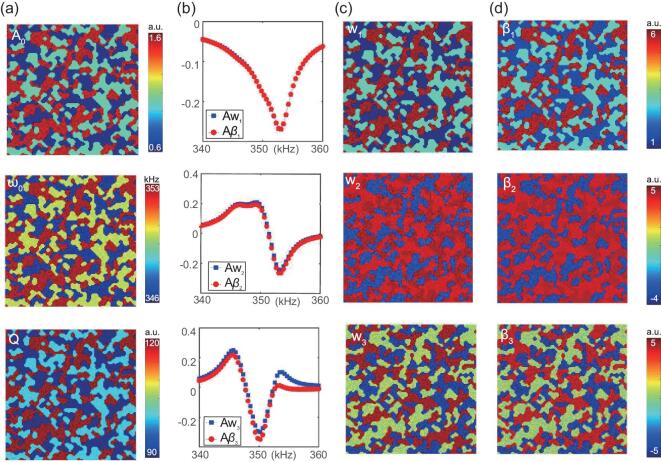
Comparison of PCA and SHO expansion for a three-phase model system with distributions of intrinsic amplitude, resonant frequency and quality factor specified in (a), from which the SE-PFM mappings can be constructed based on SHO; (b) comparison of first three spectral eigenvectors of PCA and corresponding SHO spectral basis; (c) the first three spatial eigenvectors of PCA; (d) corresponding SHO spatial basis.

The spatial variation of intrinsic amplitude, resonant frequency and quality factor—key material parameters of interest in this analysis—are not known in advance. Thus, in order to unambiguously establish our physical interpretation of PCA, we construct a model three-phase system numerically with pre-determined distribution of intrinsic and uniform amplitude, resonant frequency and quality factor for each phase, as shown in Fig. 5a, from which corresponding SE-PFM mappings can be computed using an SHO model followed by PCA analysis. Taylor expansion of SHO can then be carried out. The comparisons of the first three spectral eigenvectors are shown in Fig. 5b, which agree with each other well. Meanwhile, the comparison of spatial eigenvectors for PCA (Fig. 5c) and SHO expansion (Fig. 5d) reveals a structural similarity of over 99.9% and a Pearson correlation coefficient of over 99.8% for the first three modes, suggesting that the first three PCA spatial eigenvectors are ${{{\bf \beta }}_1}$, ${{{\bf \beta }}_2}$ and ${{{\bf \beta }}_3}$ derived from $({{{\bf \alpha }}_1}{\rm{,}}\,{{{\bf \alpha }}_2}{\rm{,}}\,{{{\bf \alpha }}_3}) = {{{\bf A}}_{{\bf 0}}}{{\bf Q}}{{{\bf \omega }}_{{\bf 0}}} \circ {\rm{[}}{{\bf 1}}{\rm{, }}{{\bf Q}} - {{\bf \bar{Q}}}{\rm{, }}\,{{{\bf \omega }}_{{\bf 0}}} - {{{\bf \bar{\omega }}}_{{\bf 0}}}]$, respectively, since the variation of ${{\bf Q}}$dominates that of ${{{\bf \omega }}_{{\bf 0}}}$ for the model system considered. This set of studies thus confirms the physical interpretation of PCA modes, which can be used to substantially speed up the analysis.

## CONCLUSIONS

Principal component analysis (PCA) has been widely used to compress and visualize multi-dimensional datasets, though its physical meaning is often unclear. SPM measures a wide range of sample properties through tip-sample interactions in terms of cantilever dynamics, though the intrinsic response is rather challenging to determine quantitatively, often interfered with various forms of crosstalk. The SE technique that we developed, in combination with a dynamics-based SHO model and data-analytic PCA, allows us to overcome such difficulties through deep data methodology. Of particular interest is a clear mechanistic understanding of the unsupervised PCA acquired through a sound physical principle, making it possible to speed up physical analysis by at least four orders of magnitude. The method can be easily implemented in any standard AFM without the need for any additional hardware and instrumentation. While the technique is demonstrated in terms of electromechanical coupling via PFM, the dynamics involved are universal in SPM, making the method applicable to improving other SPM techniques, such as in Kelvin probe force microscopy, to determine the surface potential, and in a contact resonance method to map the Young's modulus, among others.

## Supplementary Material

Supplementary Files
