# Supplementary material for: Mapping intrinsic electromechanical responses at the nanoscale via sequential excitation scanning probe microscopy empowered by deep data
Source: Natl Sci Rev. 2018 Sep 8;6(1):55–63. doi: 10.1093/nsr/nwy096 (PMC8291420; doi:10.1093/nsr/nwy096)
Supplement: Supplementary Files [file nwy096_supplemental_files.zip › SI_PCA_20180706_revising_Supplementary_File.docx]

**Mapping Intrinsic Electromechanical Responses at the Nanoscale via Sequential Excitation Scanning Probe Microscopy Empowered by Deep Data**

**Supporting Information**

**Methods**

*DART PFM*

An Asylum Research (AR) Cypher AFM was used to perform DART PFM measurements. An AC bias with an amplitude of 3 V near the sample–probe resonance frequency was applied to the PZT disk to amplify the piezoresponse via a Nanosensors PPP-EFM with a spring constant of 2.6 Nm-1. The line scan rate was set to 0.57 Hz to ensure the DART technique work smoothly.

*SE-PFM*

A series of single frequency PFM mappings were acquired under an AC voltage of 3 V and excitation frequency ranging from 320 to 400 kHz with a 2 kHz increment, as determined from a preliminary DART PFM scan that surveys the distribution of resonance frequency (ω0) over scanned region, so that the adopted frequency range covers the resonant frequencies of all the points. The scan rate was set as 1.5 Hz. Therefore, a total of 41 PFM mappings were obtained by using the programmable function Macrobuilder of AR software. All raw amplitude data were then imported into MATLAB as 41 256×256 matrices. Subpixel image registration were performed for all images by cross-correlation to correct for inevitable topography drifts during the experiment.

*Image registration*

We take the deflection mapping of the first scan as a benchmark to do the image registration for other scans. The obtained translation is applied to the amplitude and phase mappings as well to correct drifting, and we usually have to cut about 10% area on the periphery to get the overlapping area. An open-source algorithm is adopted in this process, which obtains an initial translation estimation of the cross-correlation peak and refines it by a matrix-multiply discrete Fourier transform (DFT) [1]. **Mov. S1** compares the original deflection map with the registered mappings of deflection, amplitude and phase, where each frame represents data acquired under a specific frequency.

*SHO Fitting and R2 Mapping*

Default Fit function with nonlinear least-squares method in MATLAB is used to perform SHO fitting, which returns the mappings of *A*0, *ω*0, *Q*, and *R*2. *R*2 is a statistical measure of how close the data are to the fitted regression line. It ranges from 0 to 1. *R*2=1 indicates that the fitted data explains all variability in observed data , while *R*2=0 indicates no 'linear' relationship between them. The most general definition of *R*2 is

,

where , .

*PCA Analysis*

PCA function based on SVD in MATLAB is used to perform this analysis. The variance data plotted in Fig.S3, which are also known as the eigenvalues of the covariance matrix, are from an output vector “latent” of PCA function.

*Structural similarity*

SSIM is an index for measuring the similarity between two images, which is defined as the mean of the local SSIM value map:

,

where are the average, variance, and covariance of 4×4 windows a and b that are centered in the pixel (*x*, *y*) of two images. are two variables to stabilize the division with weak denominator.

*Pearson correlation coefficient*

PCC is a measurement of the linear correlation between two vectors X and Y, which are reshaped from two images, respectively. It ranges from +1 and −1, where 1 means total positive linear correlation, 0 means no linear correlation, and −1 means total negative linear correlation,

where is the covariance and are the standard deviation of X and Y.

**Derivation of PCA and SHO on spatial mode**

*PCA modes*

Under SVD, we have , where is a diagonal matrix of positive singular values . The columns of **U** and **W** are left- and right-singular vectors and , respectively. Since unitary matrices **U** and **W** satisfy , we have

, (S1)

where are defined as PCA spatial eigenvectors sorted in the order of decreasing .

*SHO modes*

For any given pixel scanned at a specific frequency , can be reformulated as follows by plugging Eq. (1.2) into Eq. (1.1),

, (S2)

where . Then it can be expanded into 2D Taylor series around the spatial average  of the whole scanned area as,

… (S3)

where are coefficients that only depends on since are constant. This expansion can be further generalized to the whole map as,

… (S4)

where and is the Hadamard product of 1D vectors , , and that are reshaped from intrinsic parameter mappings , , and , respectively. is a 1D vector, all elements of which are 1, while and .

**Derivation of PCA and SHO on frequency mode**

*PCA modes*

After switching the row and column of , we get , where now each row represents a spectrum of data spanning all excitation frequencies for a particular grid point. Consequently, the PCA of gives principal spectral modes by computing the eigenvectors of the new covariance matrix . A simple way to do this is left multiplying on the both sides of (Eq. S1),

, (S3)

so PCA spectral eigenvectors can be generated by normalizing . According to Eq. (2) and the orthogonality of , we have , which is a vector representing the weight that takes up in each scan.

*SHO modes*

Since resembles , it is expected that should be close to as well. Besides, also represents a vector weight that takes up in each scan based on Eq. (3).

**Additional Data**


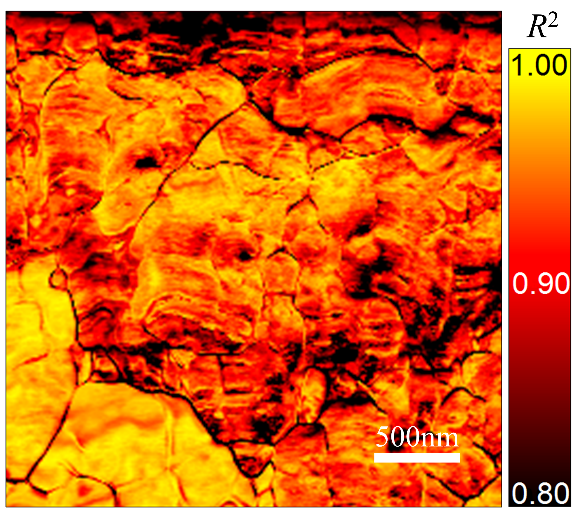


**Fig. S1** Mapping of R2


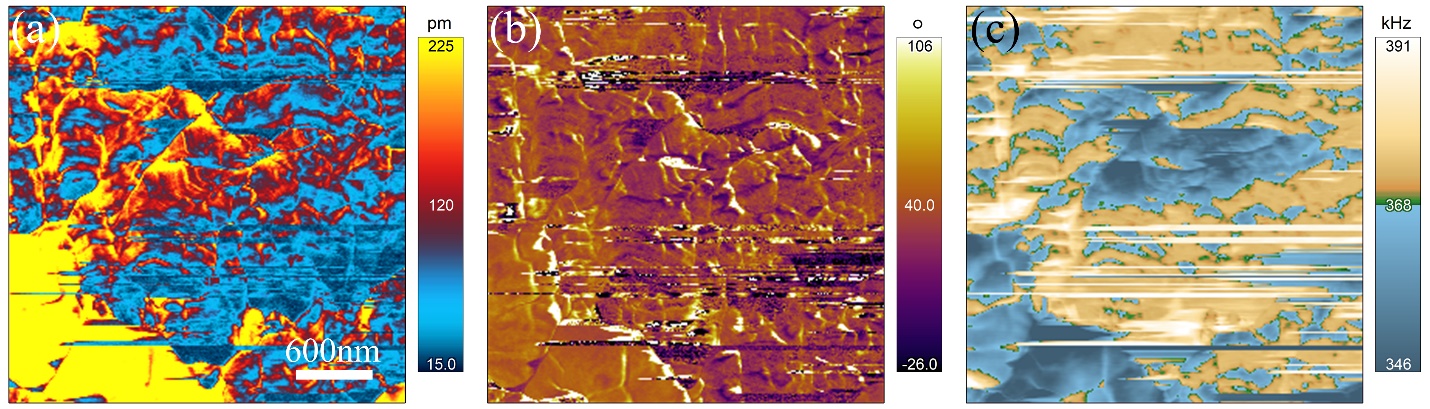


**Fig. S2** DART-PFM PZT mappings of amplitude (a) and phase (b) acquired at lower frequency of two excitation frequency (c).


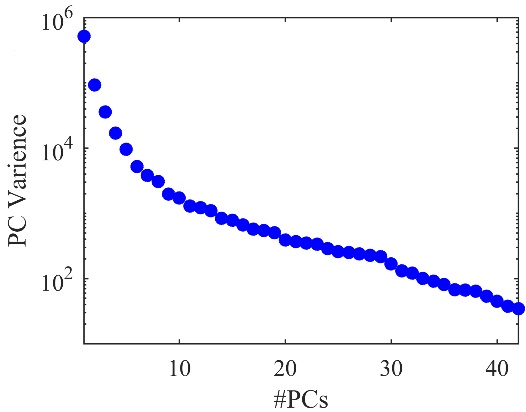


**Fig. S3** Scree plot of PCA variance for PZT.

**Mov. S1** Realignment of SE-PFM data set to correct for drifting among different scans.

**Reference:**

1. Guizar-Sicairos M, Thurman ST, Fienup JR. Efficient Subpixel Image Registration Algorithms. *Opt Lett* 2008;***33***:156–158.
